# Uterine Arteriovenous Malformation: A Rare Cause of Postpartum Hemorrhage

**DOI:** 10.7759/cureus.78730

**Published:** 2025-02-08

**Authors:** Lina Ahmad Omar Alhaj, Shadan Ahmed Falih Al-Tameemi, Tania El Hamarneh

**Affiliations:** 1 Department of Obstetrics and Gynecology, Danat Al Emarat Hospital, Abu Dhabi, ARE

**Keywords:** assisted conception, doppler ultrasound, in vitro fertilisation, in vitro fertilization ivf, mri angiography, postpartum hemorrhage, secondary postpartum hemorrhage, uterine arteriovenous malformation, uterine artery embolization

## Abstract

Uterine arteriovenous malformation (AVM) is a rare but serious cause of secondary postpartum hemorrhage (PPH). Although its overall prevalence in the general population remains uncertain, uterine AVM is recognized as an underdiagnosed condition due to its nonspecific symptoms and variable presentation. It poses significant diagnostic and therapeutic challenges, especially when presenting in the delayed postpartum period. Uterine AVM can be acquired following uterine instrumentation or trauma, leading to abnormal vascular connections within the uterine wall. This study describes a unique instance of uterine AVM identified after a cesarean section in a patient with an in vitro fertilization (IVF)-conceived pregnancy, highlighting the potential implications of assisted reproductive techniques on uterine vascular remodeling. Early recognition and multidisciplinary management ensured successful treatment, preserving the patient’s fertility and preventing severe complications.

## Introduction

Postpartum hemorrhage (PPH) remains a leading cause of maternal morbidity and mortality worldwide, particularly in the early postpartum period [1,2]. While most cases of PPH are attributed to common etiologies like uterine atony or retained placental tissue, rarer causes such as uterine arteriovenous malformation (AVM) require heightened clinical awareness [3,4].

Uterine AVM, characterized by abnormal vascular connections within the myometrium, can result in significant hemorrhage due to high-pressure blood flow through these anomalous vessels [5]. The clinical presentation of uterine AVM ranges from asymptomatic cases to severe life-threatening bleeding [4]. Diagnosis is often achieved through Doppler ultrasound and magnetic resonance imaging (MRI), which reveal characteristic vascular patterns [6]. This study presents a rare instance of uterine AVM in a 39-year-old woman following a cesarean section, emphasizing the diagnostic challenges and management strategies of this uncommon postpartum complication.

## Case presentation

A 39-year-old Filipino woman, gravida 4, para 2+2, presented with a dichorionic diamniotic twin pregnancy achieved through in vitro fertilization (IVF). Her obstetric history included an uncomplicated vaginal delivery in 2008 and two ectopic pregnancies managed surgically.

The current pregnancy was uneventful until delivery when an elective cesarean section was performed at 37+2 weeks due to the transverse lie of Twin 1. Estimated blood loss during the procedure was approximately 1 L, and mild uterine atony was successfully managed with uterotonics.

On postoperative day 11, the patient experienced sudden, severe vaginal bleeding accompanied by hemodynamic instability. Initial resuscitative measures included fluid replacement and uterotonics. Emergency dilation and curettage (D&C) were performed to evacuate any intrauterine debris, and a Bakri balloon was inserted to tamponade the bleeding, achieving temporary stabilization. Laboratory workup revealed a hemoglobin drop from 10.8 g/dL to 8.2 g/dL, necessitating blood transfusion.

**Figure 1:**
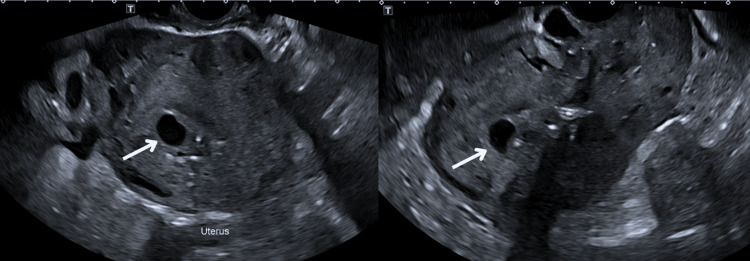
Transvaginal grayscale ultrasound of the uterus showing serpiginous anechoic spaces in the myometrium (arrows), indicating uterine AVM. AVM: arteriovenous malformation

**Figure 2:**
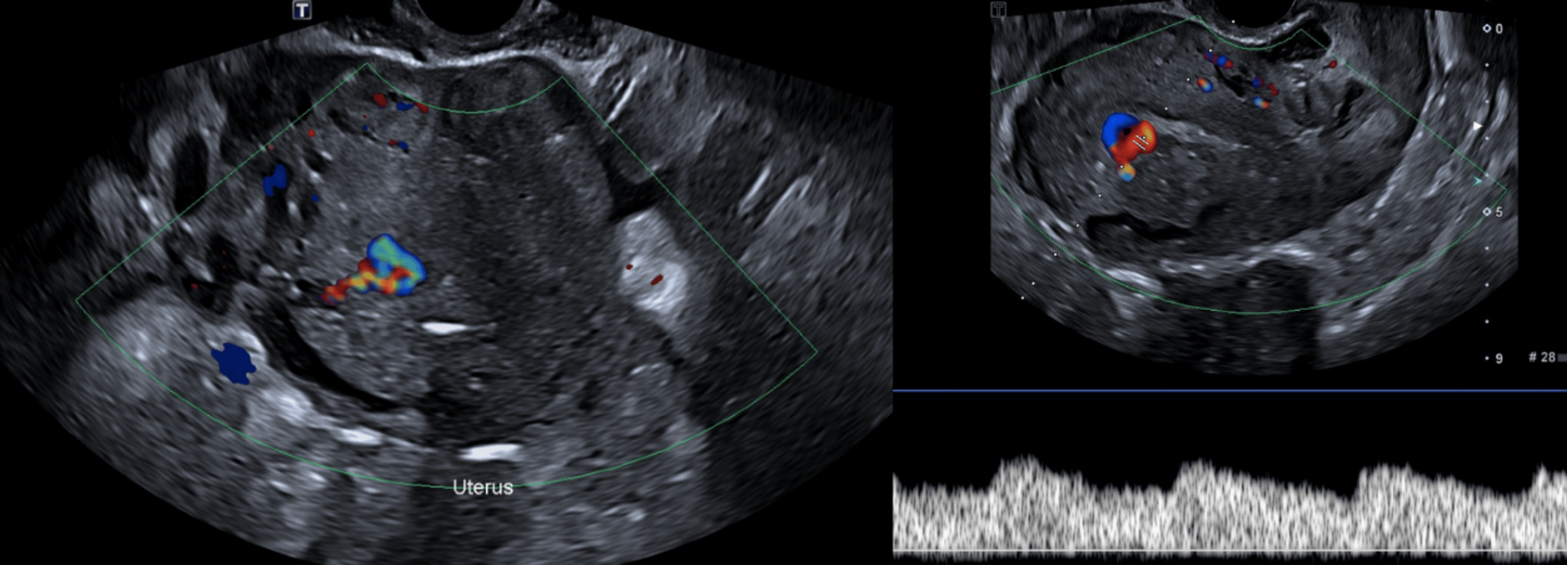
Transvaginal color Doppler ultrasound of the uterus showing turbulent flow with aliasing within the vascular channels, supporting uterine AVM diagnosis. AVM: arteriovenous malformation

**Figure 3:**
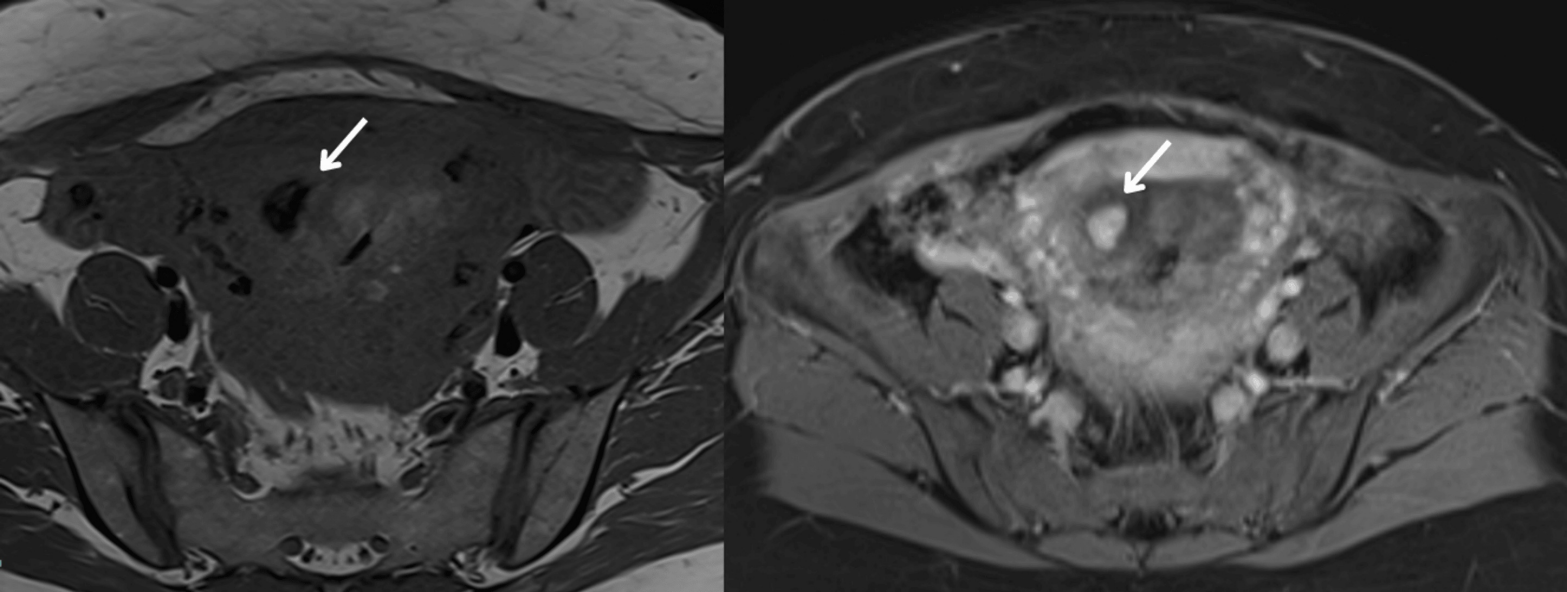
Axial T1-weighted MRI of the pelvis (pre- and post-contrast) showing serpentine myometrial vasculature (arrows) along the right lateral uterine wall with intense post-contrast enhancement, consistent with uterine AVM. AVM: arteriovenous malformation

Despite initial stabilization, the patient returned on postoperative day 15 with recurrent moderate vaginal bleeding. A thorough clinical examination ruled out uterine rupture and retained products of conception. Transvaginal Doppler ultrasound demonstrated increased vascularity with turbulent flow, raising suspicion for an AVM (Figures 1, 2). This was confirmed by MRI angiography, which revealed a prominent uterine arteriovenous fistula with no evidence of surrounding pelvic pathology (Figure 3).

A multidisciplinary team, including obstetricians, interventional radiologists, and vascular surgeons, convened to formulate a management plan. Given the patient’s desire for future fertility, uterine artery embolization (UAE) was recommended as the most appropriate intervention [7,8]. However, the patient refused the procedure and opted for conservative management with close monitoring.

The patient experienced an uneventful recovery without further bleeding episodes. At her six-week postpartum follow-up, she reported no recurrence of symptoms. Detailed counseling was provided regarding the high risks of recurrence and the implications for future pregnancies.

## Discussion

Secondary PPH, occurring 24 hours to 12 weeks postpartum, is a rare condition with an incidence of 0.21% [2]. Uterine AVM is an exceptionally rare cause, often linked to prior uterine trauma such as cesarean sections or D&C [4,6]. Early and accurate diagnosis is critical, as interventions like D&C can exacerbate an existing AVM, potentially worsening hemorrhage [4].

Diagnostic imaging, particularly Doppler ultrasound, is essential in identifying uterine AVMs, revealing hypervascularity and high systolic velocities [4]. MRI and digital subtraction angiography (DSA) provide additional detail and therapeutic options [4]. Treatment options range from conservative management to UAE and, in refractory cases, hysterectomy [4]. UAE has demonstrated high success rates, with reported efficacy reaching 93% in achieving hemostasis and resolving AVMs while preserving fertility [9]. Comparative studies also highlight UAE as a less invasive alternative with shorter recovery times and lower complication rates compared to hysterectomy, making it a preferred choice in suitable candidates [4]. UAE is favored for its high success rate and fertility preservation [4,8]. Advances in interventional radiology have further enhanced the safety and efficacy of these procedures [4,8].

In this case, the patient’s stabilization and positive response to conservative management highlight the importance of individualized treatment planning. Detailed counseling about the risks and implications of the chosen approach, including its impact on future fertility, played a critical role in ensuring patient satisfaction and adherence to the management plan. This underscores the value of shared decision-making in achieving favorable clinical and patient-centered outcomes.

## Conclusions

Uterine AVM is a rare yet significant cause of secondary PPH, often presenting diagnostic and therapeutic challenges. Early recognition, facilitated by advanced imaging modalities such as Doppler ultrasound and MRI, is essential to prevent severe morbidity and mortality. Interventional radiology plays a pivotal role in managing this condition while preserving fertility. This case underscores the importance of considering uterine AVM in patients with unexplained secondary PPH and highlights the necessity of a multidisciplinary approach to ensure optimal patient outcomes. Awareness and vigilance among clinicians can lead to timely diagnosis and improved management of this serious condition.
